# Supplementary material for: Neutralizing antibodies to SARS‐CoV‐2 Omicron variant after third mRNA vaccination in health care workers and elderly subjects
Source: Eur J Immunol. 2022 Mar 25;52(5):816–24. doi: 10.1002/eji.202149785 (PMC9087434; doi:10.1002/eji.202149785)
Supplement: Supplementary file 1 — Supplementary material [file EJI-52--s001.docx]

**Supplementary material**

**Supplementary Figure S1.** IgG concentrations expressed as BAU/ml for wild-type (WT) spike proteins (SFL and RBD) 1 month after infection with WT, Alpha or Beta variant and after 1 dose of Comirnaty COVID-19 vaccine. Each sample was tested as a technical duplicate in each experiment and the average of the two duplicates is shown. The experimental precision was confirmed by two positive control samples in each independent experiment.


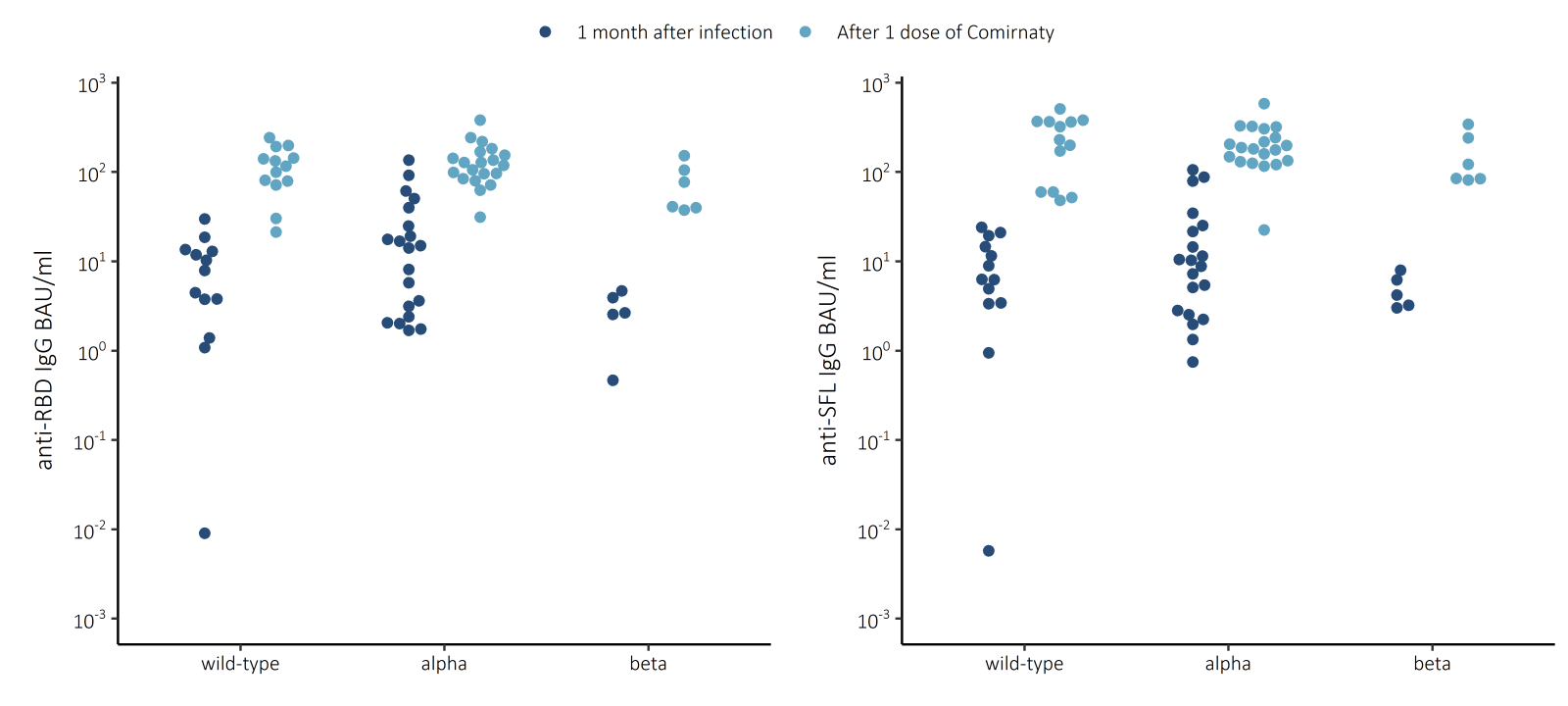


**Supplementary Table S1.** Demographic characteristics of the participants.

|  | **Age**  **Median [range]** | **Gender**  **% female** |
| --- | --- | --- |
| Health care workers (n=20) | 50.2 [27.2–63.1] | 100 |
| Elderly  (n=9) | 84.2 [71.5–89.6] | 44 |
| Infected (Wild-type)  (n=13) | 54.5 [44.7–80.8] | 62 |
| Infected (Alpha)  (n=20) | 51.2 [27.4–71.4] | 55 |
| Infected (Beta)  (n=5) | 44.0 [32.7–50.5] | 40 |
